# Nitrogen gas produces less behavioural and neurophysiological excitation than carbon dioxide in mice undergoing euthanasia

**DOI:** 10.1371/journal.pone.0210818

**Published:** 2019-01-31

**Authors:** Carlotta Detotto, Sarah Isler, Martin Wehrle, Alexei L. Vyssotski, Regula Bettschart-Wolfensberger, Thomas C. Gent

**Affiliations:** 1 Section of Anaesthesiology, Vetsuisse Faculty, University of Zürich, Zürich, Switzerland; 2 Natur- und Tierpark Goldau, Goldau, Switzerland; 3 Institute for Neuroinformatics, ETH Zürich, Zürich, Switzerland; Harvard University Faculty of Arts and Sciences, UNITED STATES

## Abstract

Carbon dioxide (CO_2_) is one of the most commonly used gas euthanasia agents in mice, despite reports of aversion and nociception. Inert gases such as nitrogen (N_2_) may be a viable alternative to carbon dioxide. Here we compared behavioural and electrophysiological reactions to CO_2_ or N_2_ at either slow fill or rapid fill in C57Bl/6 mice undergoing gas euthanasia. We found that mice euthanised with CO_2_ increased locomotor activity compared to baseline, whereas mice exposed to N_2_ decreased locomotion. Furthermore, mice exposed to CO_2_ showed significantly more vertical jumps and freezing episodes than mice exposed to N_2_. We further found that CO_2_ exposure resulted in increased theta:delta of the EEG, a measure of excitation, whereas the N_2_ decreased theta:delta. Differences in responses were not oxygen-concentration dependent. Taken together, these results demonstrate that CO_2_ increases both behavioural and electrophysiological excitation as well as producing a fear response, whereas N_2_ reduces behavioural activity and central neurological depression and may be less aversive although still produces a fear response. Further studies are required to evaluate N_2_ as a suitable euthanasia agent for mice.

## Introduction

Carbon dioxide (CO_2_) is the most commonly used gas euthanasia agent in mice due to rapid action, low cost and easy availability [1, 2]. However, multiple studies have demonstrated that carbon dioxide exposure may cause pain in humans as well as fear responses and aversion in rodents [3–6] therefore its use for euthanasia is controversial [7–12]. Alternative agents are sought to improve the welfare conditions of mice [12]. Inert gases induce unconsciousness and death by hypoxia due to the displacement of oxygen and have been investigated for humane euthanasia of rodents [13–15]. The formation of carbonic acid in the mucous membranes following CO_2_ absorption is considered to cause pain and discomfort as reported in humans [3, 16], however inert gas absorption does not result in carbonic acid formation. Furthermore, increasing acidity in the blood from CO_2_ is detected in the amygdala of mice and results in a fear response [6]. Humans undergoing short term hypoxia in hypobaric conditions do not report pain or distress and have poor perception of changes in environmental conditions [17]. Whether the same effects occur during normobaric hypoxia is uncertain. Argon (Ar) is considered to be aversive to rats [18, 19] and mice [10] presumably because loss of consciousness occurs due to hypoxia alone, and has also been documented to cause dyspnoea or “air gasping” at hypoxic conditions, which is likely to be aversive [12]. Its use is considered unsatisfactory for euthanasia, as it is slow to induce death and it causes hyperreflexia in rats [14]. Nitrogen is an inert gas which is colourless, odourless and non-irritant, but it has received little attention as a potential euthanasia agent compared to other inert gases. The proposed mechanism of N_2_-induced loss of consciousness and death is also by hypoxia [20]. One study suggested that N_2_, may be less aversive than Ar since it does not increase heart rate and mean arterial blood pressure [14]. Additionally, N_2_ gas is relatively cheap and abundant in the environment and therefore safe to the operator and non-polluting, in contrast to CO_2_, which has a significant environmental impact [21]. However, there remains insufficient data on the behavioural effects of N_2_ on mice to determine its usefulness as a euthanasia agent [20, 22].

The American Veterinary Medical Association (AVMA) euthanasia guidelines suggest an optimal fill rate between 10% and 30% chamber volume/min of CO_2_ [23]. However, these guidelines are based on extrapolation from studies performed either in other paradigms, such as comparison between pre-filled chamber and gradual induction [24], preference and approach-avoidance testing [7, 25, 26], or in other species such as rats [27], cats [28] and humans [29], without any direct pain or distress measurement specifically in mice. Recent investigations in mice have drawn different conclusions as to whether rapid (80% chamber volume/min) or slow (20–30% chamber volume/min) fill rates of CO_2_ are optimal for aversion in mice [8, 30, 31], whereas there are no reports of optimal fill rates for N_2_.

Here, we sought to determine firstly, whether N_2_ is a less aversive alternative to CO_2_ for gas euthanasia of mice, and secondly, whether slow fill is preferable to rapid fill using video tracking technology to assess behaviour (locomotion, jumping and freezing) in grouped animals. We further examined the brain activity during gas exposure in single animals chronically instrumented with EEG electrodes to test neurological excitation.

## Methods

### Animals

We used adult male (n = 135) and female (n = 119) C57Bl/6j mice (Charles Rivers Laboratories, Germany) housed in cages (Makrolon Type II long cage 365 x 205 x 140mm; Tecniplast) as litter-mates from 4 to 8 mice per cage as determined by national legislation (Tierschutzverordnung, Art. 10, Anh. 3). Cages were provided with a standard softwood bedding (Safe Lab), a piece of tissue paper (Lucart) and a resting box (Tecniplast). The median age of mice at experiment was 7.3 (6–15) weeks old and weight was 23.5 (20–25) grams. Animals were kept on a standard 12:12 light:dark cycle (lights on 08:00) and given standard rodent food (Granovit 3436) and autoclaved bottled water *ad libitum*. All experiments were performed during the light period. Animals were housed by sex from weaning at the age of three weeks and had minimal handling solely for husbandry purposes prior to experimentation. Instrumented animals were housed individually from the time of instrumentation until experimentation, in adjacent transparent cages with holes to allow diffusion of sound and smell. This was done to reduce stress associated with single housing.

### Instrumentation

To investigate brain activity associated with gas exposure, a separate cohort of animals were chronically instrumented for recording EEG and EMG (n = 6 per group). Animals were anaesthetised in isoflurane (Isoflo, Abbott, 3% volume) in oxygen (flow rate 1litre/minute) and positioned in a stereotaxic frame, as previously reported [15]. Buprenorphine (100 μg/kg; Temgesic, Schering Plough), meloxicam (5 mg/kg; Metacam, Boehringer-Ingelheim) and 0.9% saline (10 ml/kg; B. Braun) were then administered subcutaneously. The hair was then shaved from the scalp and the skin aseptically prepared, first with iodine solution (Betadine) and 4 minutes later with 70% ethanol (B. Braun). Holes were drilled in the skull and three small jewellery screws (00 x 1/8”, J.I. Morris, Southbridge, USA), soldered to 0.5mm stainless steel, PTFE wire (W3 Wire), were inserted above the dura (not penetrating brain tissue) as EEG electrodes. With respect to the cranial bregma suture, the ground electrode was placed + 4.0 mm anterior and + 1.0 mm lateral and the two recording electrodes—2.0 mm posterior and ± 2.0 mm lateral. The recorded signal was a differential voltage between the two posteriorly placed electrodes. The bare ends of two 0.5mm stainless steel, PTFE wires (W3 Wire) were implanted in the rhomboideus muscles of the neck as EMG electrodes. All electrodes were then soldered to a pin connector (M52-040023V2545, Harwin) and the implant sealed using methyl-methacrylate cement (Paladur). Animals were placed in a clean cage with fresh bedding, food and water and allowed to recover under a warming lamp (Philips). The cage was returned to the housing room when the animals were able to walk.

Animals were allowed two days to recover and were then habituated to wearing a miniature EEG recording device (Neurologger 2A; manufactured by one of the authors [32]) for 15 minutes each day for seven days whilst in the home cage. Experimentation was performed on the 9^th^ day after surgery, during the light period (08:00–20:00). Data collected from different timepoints in these experiments on some of the instrumented animals used in this study were reported in a previous study [15]. All of the animals in this investigation underwent only one gas exposure to either CO_2_ or N_2_ and one exposure to the chamber during their lifetime.

### Experiments/Recording

Experiments were performed in two separate formats. Firstly, uninstrumented animals underwent experimentation with their cage mates to assess the behaviour of animals in groups in response to CO_2_ or N_2_ exposure. Secondly, instrumented animals individually underwent experimentation to investigate the relationship between brain activity (measured by EEG) and behaviour in response to CO_2_ or N_2_ exposure.

Animals were randomly assigned to receive one of four gas exposures: CO_2_ rapid (80% chamber volume per minute; CO_2_R), CO_2_ slow (30% volume per minute; CO_2_S), N_2_ rapid (N_2_R) or N_2_ slow (N_2_S). Randomisation was performed by an automated randomisation function in Microsoft Excel (RAND() function). Briefly, the cage numbers of the animals were written in column A and a series of randomised numbers generated in column B. Column B was then sorted into descending order using the ZA↓ function, thus randomising the corresponding column A.

To prevent additional stress prior to experimentation, animals were allowed to become accustomed to the noise of the gas analyser and the experimental room whilst in the home cage for 30 minutes before starting the experiment. These measures were taken since transportation of animals the home cage itself may cause a physiological stress responses [33]. A single person was inside the experimental room to perform the experiments. Only instrumented animals were fitted with the EEG recording device on a table next to the home cage and returned to their home cage at least 30 minutes before the start of experimentation. Animals were handled solely for the purposes of fitting the EEG recording device (when appropriate) and also transfer to the chamber from the home cage which was placed immediately adjacent to it. All animals were individually lifted by their tail for transfer.

**Fig 1:**
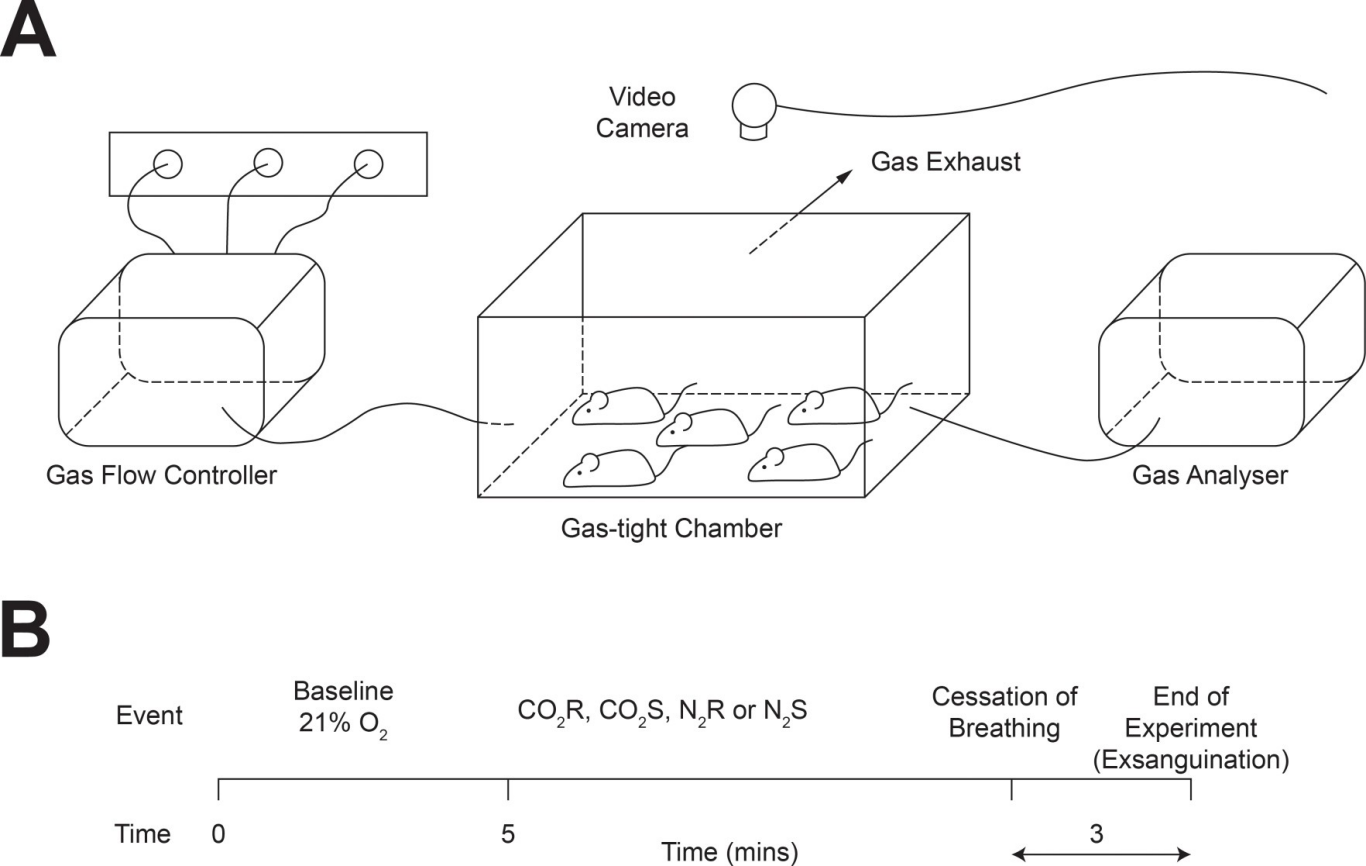
Experimental design. (A) Graphic representation of the experimental apparatus. The gas flow controller was calibrated to deliver a precise amount of each gas used and to switch from 21% oxygen to the treatment gas at the end of the baseline period. (B) Timeline of the experimental procedure. Mice were randomly assigned to one of four treatment groups: CO_2_ or N_2_ and either rapid (R—80% volume fill minute^-1^) or slow fill (S—30% volume fill minute^-1^). The experiment started with a 5-minute baseline recording at 21% O_2_. Either CO_2_ or N_2_ was then infused into the chamber as a 100% concentration of total gas inflow until cessation of breathing. The experiment was terminated three minutes after cessation of breathing and animals exsanguinated.

All experiments were performed in a non-environmentally enriched acrylic gas-tight chamber (manufactured by the authors) measuring 400 x 400 x 150 mm for uninstrumented grouped animals (Fig 1A) or 250 x 250 x 150 mm for single instrumented animals. The gas inlet and port for measuring gas concentrations were placed 25 mm from the floor of the chamber and the gas exhaust 120 mm from the roof of the chamber. Animals were naïve to the experimental chamber at the start of experimentation. Instrumented animals underwent exposure as single animals, non-instrumented animals underwent exposure in groups of littermates from one cage (4–8 animals per group).

The start of experiments consisted of a 5-minute baseline in normal air (21% oxygen, 79% nitrogen). After 5 minutes, gas exposure began with both CO_2_ and N_2_ having a concentration of 100% of total gas inflow (Fig 1B). Gas flow rates for baseline were the same as for the exposure period (i.e.: 30% or 80% chamber volume per minute) and were accurately administered by a calibrated gas mixer (GSM-3, CWE Inc, USA). Oxygen concentrations were continuously sampled, 25 mm from the floor of the chamber, during experimentation at 1L per minute and recorded electronically (Rapidox 3100EA, Cambridge Sensotec) with a sampling rate of 1Hz. Electrophysiological data was acquired at 200 Hz. The experiment was terminated 3 minutes after cessation of breathing. The chamber was cleaned with 70% ethanol (B. Braun) and allowed to air dry before the next experiment.

### Behavioural analysis

During experiments, locomotion was recorded using a video camera and analysed post hoc with video tracking software (icy Mice Profiler Tracker [34]). In the software, the number of pixels per centimetre were calculated using the known lengths of the sides of the chamber. The software then automatically detected the centre of each mouse, based on contrast to the background. The distance moved by each animal was then calculated for each second and the speed then calculated in centimetres per second. Normalised speeds were then calculated by dividing the speed during each second of the gas exposure by the average speed of the last 30 seconds of the baseline. In addition, we measured the number of vertical jumps, defined as vertical rapid jumps [35], both during baseline and gas exposure. Furthermore, we assessed freezing episodes, defined as a minimum period of two seconds of complete inactivity except that which is necessary for respiration, whilst the animal is standing with the head raised [6, 9], by visual scoring of videos throughout gas exposure. To ensure that all EEG and behavioural measurements were taken from the time when the animals were conscious, we determined the time point of loss of motion (LOM) which has been shown to occur momentarily before loss of righting reflex (LORR) [36, 37]–which is considered as a proxy for loss of consciousness in rodents [38]. This was determined from the calculated speed given by the video tracking software and visual scoring to assess that no purposeful movements were being made, such as walking, attempting to stand and grooming. LOM was considered to have occurred from the time point where locomotion speed was 0 cm/s and remained at this value for the remainder of the experiment. The number of vertical jumps, freezing episodes and LOM were scored by an observer who was blinded to the treatment of the animals. Experiments were terminated 3 minutes after the animals stopped breathing and animals were exsanguinated by cardiac puncture to ensure death. For instrumented animals, the EEG recording device then was detached from the animal and the data downloaded onto a computer.

### EEG analysis

Analysis of EEG recordings was performed in waveform analysis software (Spike 2, Cambridge Electronic Design, CED). Isoelectric EEG was defined as the point were no discernible oscillations were detectable, and was used as a marker of cessation of neocortical activity and therefore brain death [15]. EEG oscillations during slow-wave sleep and light general anaesthesia are characterised by slower rhythmic waves in the delta range (1–4.5 Hz) [38]. Whereas, wakefulness is characterised by faster theta rhythms (5–9 Hz) in the EEG. During exploratory behaviours and freezing behaviour, the frequency and power of theta oscillations further increases in the EEG of rodents [39, 40]. The power ratio between the two bands is considered to be an accurate measure of vigilance state and behavioural excitation [41, 42]. Band pass filters were applied to the raw EEG to create separate channels of theta (5–9 Hz) and delta (1–4 Hz) signals. The band passed signals were multiplied by themselves to create a signal of power (μV^2^) and theta:delta ratio calculated by dividing the theta power signal by the delta power signal, such that a value > 1 indicates a predominant theta oscillation and a value < 1 indicates a predominant delta oscillation. Theta:delta ratio was calculated in 1 second bins.

### Statistical analysis

Each animal was considered a single entity for data analysis whether housed in groups or alone. Data were analysed by one-way ANOVA with post-hoc Tukey’s modification for parametric data and Kruskal-Wallis test for non-parametric data. P-values less than 0.05 were considered significant. Values for all mice within a cage were summated for comparison between baseline and treatment. Statistical analysis was performed using Graphpad Prism 6.0. Values in the text are reported as median and range for non-parametric and mean ± s.e.m for parametric data. Locomotion data, jumps and freezing episodes were plotted as cumulative frequencies with respect to oxygen concentration. Locomotion data were normalised to activity of the last 30 seconds of the baseline. Differences in distributions were calculated using the Kolmogorov-Smirnov test. Comparisons of baseline and treatment parameters were performed by paired T-test.

### Exclusion criteria

Animals were excluded from experimentation if they showed signs of pain assessed by a grimace scale score greater than 1 (as assessed from still photographs taken from cage-side video recordings [43]) or if body weight had not returned by 3 days after surgical implantation. Segments of electrophysiological data were excluded from analysis if they contained movement artefacts, defined as single deflections of more than 400 μV lasting more than 200 ms during movement of the animal, or any segments with a saturated signal, where the recorded amplitude range was ± 500 μV.

### Ethical approval

This work was approved by the Kanton Zürich Gesundheitsdirektion Veterinäramt. License numbers: 58/2014 and 051/2016

## Results

No animals were excluded from experimentation or analysis. To determine whether nitrogen is less aversive than carbon dioxide as a euthanasia agent for mice, we first assessed the behavioural parameters of groups of non-instrumented C57Bl/6 mice being euthanised in four different treatment groups (CO_2_R; n = 10 cages; female: n = 5; male: n = 5; N_2_R; n = 10 cages; female: n = 5; male: n = 5; CO_2_S; n = 10 cages; female: n = 5; male: n = 5; and N_2_S; n = 10 cages; female: n = 5; male: n = 5). There were no significant differences in the number of animals per cage between the four gas exposure groups (CO_2_R; n = 6 (4–7); N_2_R; n = 6 (5–8); CO_2_S; n = 6 (5–8); N_2_S; n = 5.5 (4–8); P = 0.41; F = 0.99; one-way ANOVA).

### Locomotion activity

**Fig 2:**
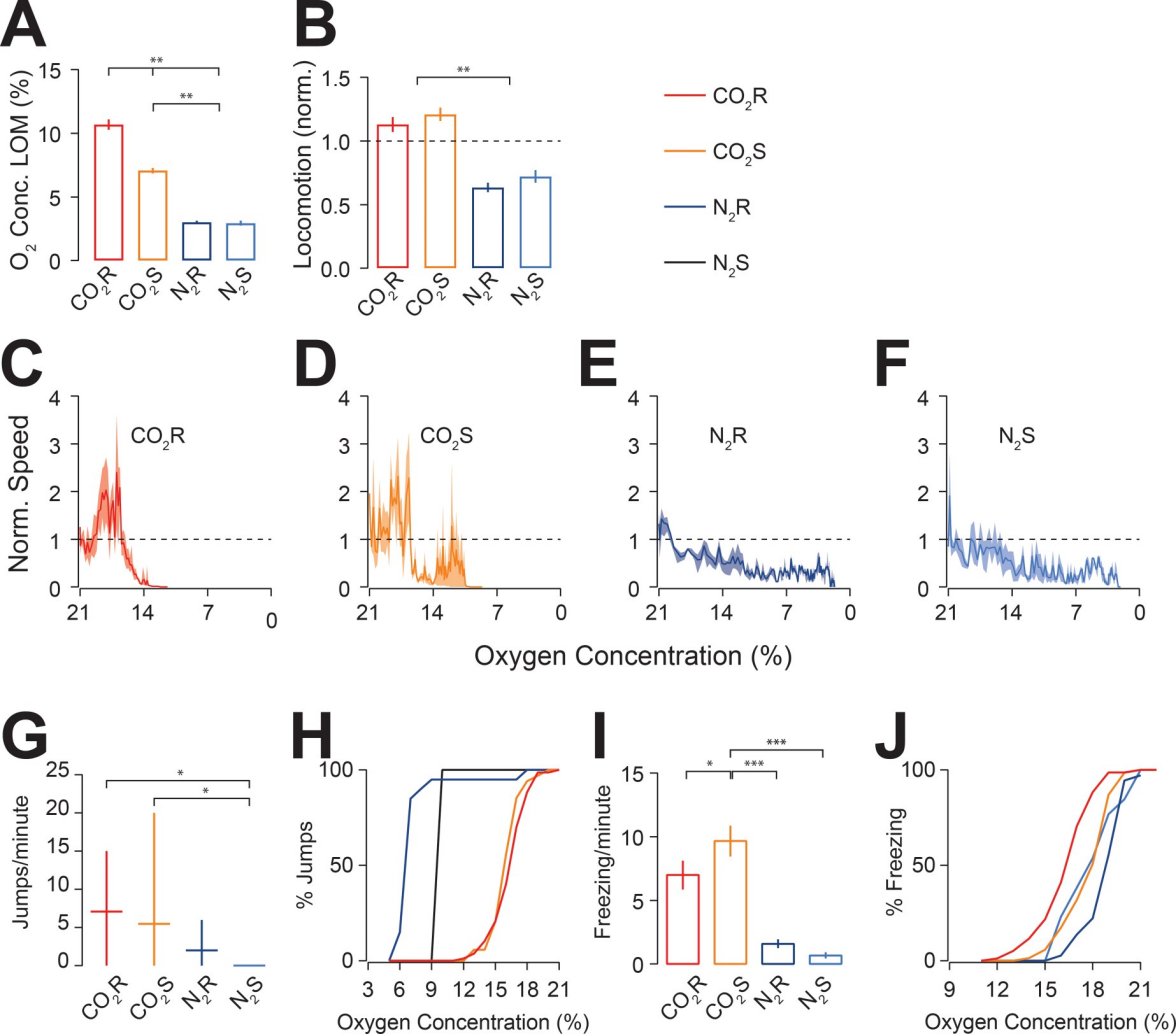
Effects of CO_2_ and N_2_ on locomotion, jumping and freezing in grouped mice. (A) Average (mean ± s.e.m) oxygen concentration at loss of motion (LOM). CO_2_R resulted in LOM at higher oxygen concentrations than other groups. CO_2_S resulted in LOM at oxygen concentrations higher than nitrogen groups (**P < 0.01). (B) Average speed (mean ± s.e.m), normalised to the last 30 seconds of baseline. Horizontal line indicates the normalisation value for baseline measurements. CO_2_ resulted in increased locomotion compared to nitrogen (**P < 0.01). (C-F) Normalised speed in relation to oxygen concentration from the start of gas exposure until LOM for CO_2_R (C), CO_2_S (D), N_2_R (E), and N_2_S (F). (G) Aversive jumps shown as median (± range) of total jumps and inter-quartile range per cage. Both CO_2_R and CO_2_S resulted in significantly more jumps compared to N_2_S (*P < 0.05) (H) Cumulative curves indicating the relative number of jumps in relation with oxygen concentration of the four treatment groups. (I) Freezing shown as mean (± s.e.m) number of freezing episodes per minute per animal. Mice in CO_2_S froze significantly more times than in the other three groups (*P < 0.05; ***P < 0.001). (J) Cumulative curves indicating the relative freezing episodes in relation with the oxygen concentration of the four treatments groups.

First, we measured locomotor activity of groups of non-instrumented mice during exposure to CO_2_ and N_2_ with either rapid or slow fill in a euthanasia paradigm. We first investigated the differences in oxygen concentration at loss of motion (LOM), defined as the point when the last animal in the group stopped purposeful movements excluding breathing, which we interpreted as the first stage towards unconsciousness. Interestingly, we found that CO_2_R resulted in LOM at higher oxygen concentrations than CO_2_S (CO_2_R: 10.7 ± 0.4%; CO_2_S: 7.1 ± 0.2%; P < 0.01; one-way T-test; Fig 2A). In comparison N_2_ groups reached LOM at significantly lower oxygen concentrations than CO_2_ (P = 0.0058; F = 5.0; one-sided ANOVA) however there was no difference between N_2_R and N_2_S groups (N_2_R: 3.0 ± 0.1%; N_2_S: 2.9 ± 0.2%; P = 0.073; one-way T-test; Fig 2A).

We then compared the speed of locomotion during gas exposure, normalised to the last 30 seconds of baseline, for each treatment group and plotted it against O_2_ concentration. These measures were therefore independent of time, allowing direct comparison of rapid and slow fill groups. We found no difference in speed between treatment groups during the last 30 s of baseline (P = 0.43; F = 0.96; one-way ANOVA). We found that there were no differences between groups for the average locomotion speed during baseline (CO_2_R: 0.84 ± 0.17 cm/s; N_2_R: 0.79 ± 0.20 cm/s; CO_2_S: 0.83 ± 0.23 cm/s; N_2_S: 0.81 ± 0.11 cm/s; P = 0.97; F = 0.015; one-way ANOVA). We found that there was a significant increase in locomotion speed for both CO_2_R (ratio of baseline locomotion and gas exposure locomotion: 1.13 ± 0.06; P = 0.028, t = 2.25; two-sided T-test) and CO_2_S (1.21 ± 0.05, P = 0.002; t = 3.97; two-sided T-test) groups compared to baseline during the first 30s of exposure. Interestingly, we found a significant decrease of activity for both N_2_R (0.64 ± 0.04; P < 0.0001; t = 9.77; two-sided T-test) and N_2_S (0.72 ± 0.05; P < 0.0001; t = 5.69; two-sided T-test) groups (Fig 2B). We found no significant difference in locomotion speed between rapid and slow groups for either CO_2_ (P > 0.05; two-sided T-test) or N_2_ (P > 0.05; two-sided T-test). We found no difference in locomotion speed between males and females within the different groups (P > 0.05; one-way ANOVA). To determine the effect of gas exposure on locomotion, we compared the speed of locomotion between groups from the start of exposure until LOM and normalised to oxygen concentration (Fig 2C–2F). We found that in both CO_2_ groups, there was a transient spike in speed followed by a rapid decline, which followed the same distribution, irrespective of oxygen concentration (P = 0.11; Kolmogorov-Smirnov test). Interestingly, for CO_2_S at lower oxygen concentrations, there was a sudden increase in activity not seen with CO_2_R (P < 0.05; Kolmogorov-Smirnov test). In contrast, speed declined slowly for both N_2_ treatments with no significant differences between groups (P > 0.05; Kolmogorov-Smirnov test).

### Jumps

Vertical jumping in mice is considered to be a behavioural response to an aversive environment, independent of pain [44] and has been reported previously in response to CO_2_ exposure, interpreted as aversive behaviour [35]. Therefore, we measured the rate of vertical jumps during gas exposure in non-instrumented mice and compared them to baseline conditions as a measure of aversion. We did not observe any other forms of jump, such as forward leaping. We found that jumps were commonly shown and occurred significantly more often when compared to baseline for CO_2_R (0.036 ± 0.08 jumps min^-1^, P = 0.0063; two-sided T-test) and CO_2_S (0.03 ± 0.01 jumps min^-1^; P = 0.0037; two-sided T-test) groups, but not N_2_R (P = 0.11; two-sided T-test) or N_2_S (P = 0.34; two-sided T-test). Indeed, both CO_2_R and CO_2_S resulted in significantly more jumps than N_2_S (0 jumps; P = 0.0056; P = 0.0353; respectively one-way ANOVA). However, there was no significant difference between the CO_2_S and N_2_R (0 jumps; P > 0.05, one-way ANOVA), mice exposed to CO_2_R showed significantly more jumps per minute that in N_2_R group (P = 0.0305, one-way ANOVA; Fig 2G). No jumps were observed during baseline conditions. To determine the effect of gas exposure on vertical jumps, we compared the occurrence of jumps between groups from the start of exposure until LOM with respect to oxygen concentration (Fig 2H). We found no difference in distribution between treatment CO_2_ groups (P = 0.35; Kolmogorov-Smirnov test) or between N_2_ groups (P = 0.10; Kolmogorov-Smirnov test). However, we found that jumps occurred at lower oxygen concentrations for N_2_ groups compared to CO_2_ (P < 0.01; Kolmogorov-Smirnov test).

### Freezing

CO_2_ has previously been shown to cause fear responses [6], which can be assessed by freezing behaviour, an immobile state without any movements except respiration whilst the animal is standing with the head raised [6, 9]. Therefore, we investigated if fear responses were a component of the increased aversion by measuring freezing behaviour during gas exposure. We found that mice did not freeze during baseline. Mice in CO_2_S (9.7 ± 1.2 freezing episodes/minute) froze significantly more often than CO_2_R (7.0 ± 1.1 freezing episodes/minute; P = 0.012; two-sided T-test), N_2_R (1.6 ± 1.4 freezing episodes/minute; P < 0.001; two-sided T-test) and N_2_S (0.7 ± 0.2 freezing episodes/minute P < 0.001; two-sided T-test; Fig 2I). We found no differences between N_2_ groups (P > 0.05; two-sided T-test). No difference in freezing time was observed (CO_2_R: 2.6 ± 0.2 s; CO_2_S: 2.8 ± 0.1 s; N_2_R: 2.5 ± 0.2 s; N_2_S: 2.7 ± 0.3 s; P = 0.44; One-way ANOVA; Data not shown). We did not observe ataxia during the period when animals exhibited freezing behaviour. Interestingly, there was no difference between groups in oxygen concentration for the occurrence of freezing episodes (P > 0.05; Kolmogorov-Smirnov test; Fig 2J).

### Electrocortical activity

**Fig 3:**
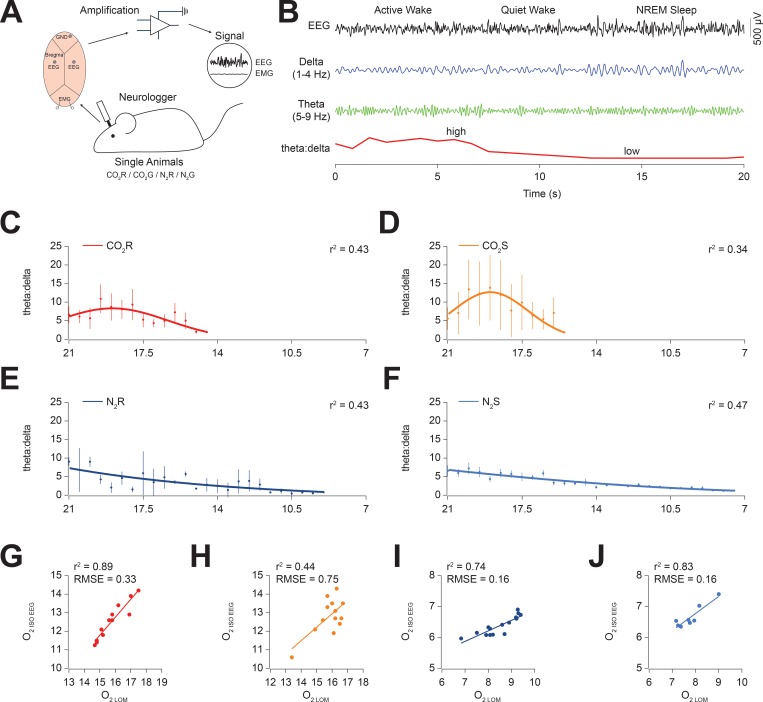
CO_2_ causes central neurological excitation whereas N_2_ causes central neurological depression. (A) Schematic of recording. From bottom going clockwise: Single instrumented mice were used and underwent euthanasia with CO_2_ or N_2_ at either rapid fill (R—80% volume fill minute^-1^) or slow fill (G—30% volume fill minute^-1^). EEG and EMG electrodes were chronically implanted and animals allowed to recover for seven days. Data were acquired on a portable wireless Neurologger at 200 Hz and data downloaded after the experiment onto a computer. (B) Representative sample of a 20 s EEG recording from a mouse recorded during baseline. Filtered delta (1–4 Hz; blue) and theta (5–9 Hz; green) signals are shown below. Note the difference in amplitude between the delta and theta signals changing over time. The theta:delta power ratio (TD) shown in red on the bottom line. Note the high TD when theta amplitude is high and low TD when delta amplitude is high. (C, D, E, F) TD values with respect to O_2_ concentration for CO_2_R (C), CO_2_S (D), N_2_R (E) and N_2_S (F). Note the initial increase in TD for CO_2_ groups compared to the slow reduction in N_2_ groups. (G, H, I, J) Linear regressions of O_2_ concentration at isoelectric EEG (ISO-EEG) against O_2_ concentration at LOM for CO_2_R (G), CO_2_S (H), N_2_R (I) and N_2_S (J). Note the increased scatter of points for CO_2_S.

Unlike CO_2_, N_2_ does not have true narcotic effects at normobaric conditions, instead it produces unconsciousness and death by hypoxia. Given that hypoxia could also cause muscle weakness and ataxia we sought to determine whether the reduced locomotion and behaviour was due to central sedative effects of the hypoxia or peripheral muscle weakness. Therefore, we recorded EEG/EMG in individual chronically instrumented mice during the euthanasia paradigm (Fig 3A; CO2R: n = 8 animals; CO2S: n = 10 animals; N2R: n = 9 animals; N2S: n = 10 animals).

In single instrumented animals, we found behavioural patterns (locomotion, jumping and freezing) which paralleled our measurements from grouped animals, suggesting that behaviours were not affected by the animal carrying the Neurologger (S1 Fig). We measured the ratio of theta and delta (TD) oscillations in the EEG, as a measure of neocortical excitation during gas exposure (Fig 3B; ref: [45]). We found that in CO_2_ groups there was a significant increase in TD compared to baseline (P = 0.015; F = 4.1; one-way ANOVA; Fig 3C and 3D) suggesting heightened central neuronal arousal. Baseline values of TD were not significantly different between groups (CO2R: 6.6 ± 2.3; CO2S: 5.6 ± 0.9; N2R: 8.1 ± 1.1; N2S: 6.5 ± 1.5; P = 0.61; F = 0.61; one-way ANOVA). Interestingly, TD increased significantly more for CO_2_S compared to CO_2_R (measured at the curve apex; CO2R: 8.3 ± 1.6; CO2S: 12.7 ± 1.3; P = 0.048; t = 2.15; d.f. = 15; two-sided T-Test). By contrast, TD decreased uniformly from baseline, inversely proportional to O_2_ concentration in N_2_ groups (P = 0.015; F = 4.1; one-way ANOVA; Fig 3E and 3F). This data suggests that mice in N_2_ groups experienced central neuronal depression as a result of decreasing O_2_ concentration. Given the different O_2_ concentrations of LOM between CO_2_ and N_2_ groups, we used the EEG to determine O_2_ concentrations at the time of brain death determined by isoelectric EEG (ISO-EEG). We then compared these values to O_2_ concentrations at LOM as a measure of the reliability of the euthanasia method. As expected, we found that brain death occurred at higher O_2_ concentrations for CO_2_ groups compared to N_2_ groups (CO2R: 12.6 ± 0.3; CO2S: 12.8 ± 2.7; N2R: 6.4 ± 0.1; N2S: 6.7 ± 0.1; P < 0.0001; F = 286.3; one-way ANOVA). Interestingly, we found a high correlation between LOM and ISO-EEG for CO_2_R (r^2^ = 0.89), N_2_R (r^2^ = 0.74) and N_2_S (r^2^ = 0.83) groups but low correlation for CO_2_S (r^2^ = 0.44). Furthermore, the fits were significantly different between CO_2_ groups, as measured from the root mean square errors (RMSE; CO_2_R: 033; CO_2_S: 0.75; P = 0.03; *t* = 2.3; d.f. = 23; two-way T-test) suggesting less predictability for the point of death when using CO_2_S.

## Discussion

The AVMA guidelines state that the use of humane euthanasia techniques should induce the most rapid, painless and distress-free death as possible [23]. Identifying pain during mice euthanasia is a critical end-point and currently, no objective measures have been validated for assessing pain associated with euthanasia [30]. However previous studies have identified markers of aversion [35] and fear [6] in response to CO_2_ exposure. Since inert gases are colourless, odourless and non-irritant, we hypothesised that N_2_ would be less aversive than CO_2_ in a gas euthanasia paradigm.

We found that mice exposed to CO_2_ showed behavioural excitation, manifested by increased locomotion, freezing and jumping compared to baseline, which others have interpreted as signs of aversion [11, 25, 35, 46]. Thomas et al., [35] previously reported jumping in response to CO_2_ exposure which the authors interpreted as escape behaviour. Based on an observation of exaggerated jumping with the addition of N_2_, they hypothesised that jumping resulted from hypoxia. However, in our study, we noted more numerous jumps with CO_2_ compared to N_2_, and at higher O_2_ concentrations, suggesting that the jumps observed with CO_2_ are primarily a result of CO_2_ exposure, although hypoxia may also cause jumping [35]. This aversion may be due to carbonic acid formation in the nasal mucous membranes causing irritation [6, 47]. Additional brainstem circuits have been identified which produce arousal from natural sleep in response to CO_2_ [48]. These identified neurones in the parabrachial nucleus are strong modulators of neocortical excitation [49]. Thus, there are multiple potential mechanisms for producing the neurological and behavioural excitation that we observed. However, both CO_2_ and hypoxia are likely to be factors in inducing this behaviour. Interestingly, they also noted a decreased jumping with the addition of N_2_O to CO_2_ due to an increased systemic absorption of CO_2_ [35]. We did not observe reduced jumping with rapid fill carbon dioxide but we cannot quantitatively compare absorption rates in the two studies. However, we did observe that CO_2_S resulted in increased freezing compared to CO_2_R, suggesting a fear response to its exposure [6, 9]. Furthermore, we showed heightened excitation of brain activity to slow fill CO_2_ compared to rapid fill, suggesting that the duration of exposure to CO_2_ might be a more important factor than the concentration of CO_2_ itself for behavioural and neurological excitation. Interestingly, we observed no jumping or freezing behaviour during baseline activity. However, other strains of mice that show increased stress responses compared to C57Bl6 mice [50] might exhibit this behaviour during the baseline.

The AVMA guidelines report that “an optimal flow rate for CO_2_ euthanasia systems should displace 10% to 30% of the chamber or cage volume/min”, however the use of CO_2_ at high flow is not specifically contraindicated in the AVMA recommendations. Rather, it is considered unacceptable to place a conscious animal inside a 100% CO_2_ pre-filled chamber [23]. Previous investigation into different fill rates of CO_2_ concluded that slow fill did not lessen behavioural, cardiovascular or histological markers of distress in mice [30]. Taken together, these data suggest that use of rapid fill CO_2_ may result in less aversion than slow fill. Indeed, rapid fill might be beneficial compared to slow fill, which may cause a longer time until loss of consciousness and death, and prolonged period of dyspnoea, anxiety or distress [12, 30].

Conversely, mice exposed to N_2_ decreased their locomotor activity compared to baseline, suggesting a sedative effect of reduced atmospheric oxygen, as suggested by previous work [51]. Reduced locomotion speed did not result from prolonged freezing, since freezing episode duration was the same between groups. We further found that behavioural excitation or retardation was mirrored by neurophysiological activity. Based on behavioural assessment alone, one might surmise that reduced locomotion and jumping during N_2_ exposure might result from muscle weakness induced by hypoxia. However, we showed that brain activity depression occurs at the same time, raising the likelihood that the reduced behaviour is mediated by brain activity. However, a direct causality remains to be determined. Hypoxia in mice causes air hunger [35] as well as in other species [19]. Although we did not measure respiratory values in our study, other behavioural and electrophysiological variables were low at hypoxic conditions for animals exposed to N_2_. The impact of air hunger on animal welfare in this context requires further evaluation. Furthermore, freezing was not dependent on oxygen concentration between groups and therefore is unlikely to be due to hypoxia in the N_2_ groups, although previous reports of argon induced hypoxia showed that it was aversive to mice [10], which would be consistent with our results.

Exposure to CO2 is reported to result in pain in humans [3, 16] which is assumed to occur in rodents as well. Pain was not an endpoint for our study and is considered difficult to gauge in mice during acute conditions [20]. Based on these studies documenting pain and irritation following CO_2_ exposure in humans, it is likely that mice in this study also experienced pain, although this cannot be directly assessed. The degree of distress from this pain results from the pain intensity and its duration. In that regards, slow fill regimes in which animals are exposed to CO_2_ for longer are likely to result in more distress, although this requires further investigation. Hypoxia also causes pain manifested as headache in humans [52], although this is typically associated with less severe hypoxia than used in this study. Furthermore, the onset of pain takes longer, in the region of several hours. Whether pain manifests during very short-term hypoxia as experienced in the N_2_ groups remains to be determined.

Our results show that CO_2_ slow fill does not prevent aversive behaviour such as jumping or increased locomotion and moreover it caused more freezing episodes than rapid fill CO_2_. The decreased incidence of freezing during rapid fill CO_2_ indicates a low concentration threshold for the fear component of CO_2_ exposure, as previously reported [6]. Therefore, rapid fill would reduce the time from this threshold until a hypnotic/narcotic effect, therefore potentially reducing the number of jumps and freezing episodes observed. This would be supported further by our observation that freezing was not dependent on fill rate. A significant proportion of freezing from CO_2_ exposure is a chemical effect, as indicated by the lower incidence in nitrogen groups. Furthermore, mice showed increased locomotor activity at lower oxygen concentrations in the CO_2_ slow fill group, suggesting that this increases the likelihood of animals becoming hypoxic before losing consciousness.

## Conclusion

Our study demonstrates that N_2_ produces reduced behavioural and electrophysiological excitation during euthanasia compared to CO_2_. However, N2 may still cause some degree of fear response. Furthermore, rapid fill CO_2_ might be less aversive than slow fill. Further work is required to assess other physiological and behavioural parameters, such as nociception, to determine if N_2_ is a suitable euthanasia agent for mice.

## Supporting information

S1 Fig Effects of CO_2_ and N_2_ on locomotion, jumping and freezing in single instrumented mice. Behavioural markers in the four treatment groups: CO_2_ rapid fill (CO_2_R), CO_2_ slow fill (CO_2_S), nitrogen rapid fill (N_2_R) and nitrogen slow fill (N_2_S). (A) Average (mean ± s.e.m) oxygen concentration at loss of motion (LOM). CO_2_R resulted in LOM at higher oxygen concentrations than other groups. CO_2_S resulted in LOM at oxygen concentrations higher than nitrogen groups (**P < 0.01; ****P < 0.0001). (B) Average speed (mean ± s.e.m), normalised to the last 30 seconds of baseline. Horizontal line indicates the normalisation value for baseline measurements. CO_2_ resulted in increased locomotion compared to nitrogen (**P < 0.01). (C-F) Normalised speed with respect to oxygen concentration from the start of gas exposure until LOM for CO_2_R (C), CO_2_S (D), N_2_R (E), and N_2_S (F). (G) Vertical jumps shown as median of jumps per animal and inter-quartile range per cage. Both CO_2_R and CO_2_S resulted in significantly more jumps compared to N_2_S (***P < 0.001; ****P < O.0001) (H) Cumulative curves indicating the relative number of jumps in relation with oxygen concentration of the four treatment groups. (I) Freezing shown as mean (± s.e.m) number of freezing episodes per minute per animal. Mice in CO_2_S froze significantly more times than in the other three groups (**P < 0.01). (J) Cumulative curves indicating the relative freezing episodes in relation with the oxygen concentration of the four treatment groups.
(TIF)

## References

[1] Artwohl J, Brown P, Corning B, Stein S, Force AT. Report of the ACLAM Task Force on Rodent Euthanasia. J Am Assoc Lab Anim Sci. 2006;45(1):98–105. . 16548095

[2] Valentim AM, Guedes SR, Pereira AM, Antunes LM. Euthanasia using gaseous agents in laboratory rodents. Lab Anim. 2015 10.1177/0023677215618618 . 26609130

[3] Danneman PJ, Stein S, Walshaw SO. Humane and practical implications of using carbon dioxide mixed with oxygen for anesthesia or euthanasia of rats. Lab Anim Sci. 1997;47(4):376–85. . 9306311

[4] Conlee KM, Stephens ML, Rowan AN, King LA. Carbon dioxide for euthanasia: concerns regarding pain and distress, with special reference to mice and rats. Lab Anim. 2005;39(2):137–61. 10.1258/0023677053739747 . 15901358

[5] Valentine H, Williams WO, Maurer KJ. Sedation or inhalant anesthesia before euthanasia with CO2 does not reduce behavioral or physiologic signs of pain and stress in mice. J Am Assoc Lab Anim Sci. 2012;51(1):50–7. Epub 2012/02/15. . 22330868 PMC3276966

[6] Ziemann AE, Allen JE, Dahdaleh NS, Drebot II, Coryell MW, Wunsch AM, et al The amygdala is a chemosensor that detects carbon dioxide and acidosis to elicit fear behavior. Cell. 2009;139(5):1012–21. Epub 2009/12/01. S0092-8674(09)01355-5 [pii] 10.1016/j.cell.2009.10.029 . 19945383 PMC2808123

[7] Leach MC, Bowell VA, Allan TF, Morton DB. Measurement of aversion to determine humane methods of anaesthesia and euthanasia. Animal Welfare. 2004;13:S77–86.

[8] Moody CM, Chua B, Weary DM. The effect of carbon dioxide flow rate on the euthanasia of laboratory mice. Lab Anim. 2014;48(4):298–304. Epub 2014/08/07. 0023677214546509 [pii] 10.1177/0023677214546509 . 25097256

[9] Mongeluzi DL, Rosellini RA, Ley R, Caldarone BJ, Stock HS. The conditioning of dyspneic suffocation fear. Effects of carbon dioxide concentration on behavioral freezing and analgesia. Behav Modif. 2003;27(5):620–36. 10.1177/0145445503256316 . 14531158

[10] Makowska IJ, Vickers L, Mancell J, Weary DM. Evaluating methods of gas euthanasia for laboratory mice. Appl Anim Behav Sci. 2009;121(3–4):230–5. 10.1016/j.applanim.2009.10.001 WOS:000272977500012.

[11] Leach MC, Bowell VA, Allan TF, Morton DB. Aversion to gaseous euthanasia agents in rats and mice. Comp Med. 2002;52(3):249–57. Epub 2002/07/10. . 12102571

[12] Hawkins P, Prescott MJ, Carbone L, Dennison N, Johnson C, Makowska IJ, et al A Good Death? Report of the Second Newcastle Meeting on Laboratory Animal Euthanasia. Animals (Basel). 2016;6(9). 10.3390/ani6090050 . 27563926 PMC5035945

[13] Burkholder TH, Niel L, Weed JL, Brinster LR, Bacher JD, Foltz CJ. Comparison of carbon dioxide and argon euthanasia: effects on behavior, heart rate, and respiratory lesions in rats. J Am Assoc Lab Anim Sci. 2010;49(4):448–53. Epub 2010/09/08. . 20819391 PMC2919185

[14] Sharp J, Azar T, Lawson D. Comparison of carbon dioxide, argon, and nitrogen for inducing unconsciousness or euthanasia of rats. J Am Assoc Lab Anim Sci. 2006;45(2):21–5. . 16542038

[15] Gent TC, Detotto C, Vyssotski AL, Bettschart-Wolfensberger R. Epileptiform activity during inert gas euthanasia of mice. PLoS One. 2018;13(4):e0195872 10.1371/journal.pone.0195872 . 29672545 PMC5908136

[16] Wise PM, Wysocki CJ, Radil T. Time-intensity ratings of nasal irritation from carbon dioxide. Chem Senses. 2003;28(9):751–60. . 14654442 10.1093/chemse/bjg065

[17] Neuhaus C, Hinkelbein J. Cognitive responses to hypobaric hypoxia: implications for aviation training. Psychol Res Behav Manag. 2014;7:297–302. 10.2147/PRBM.S51844 25419162 PMC4234165

[18] Niel L, Weary DM. Rats avoid exposure to carbon dioxide and argon. Appl Anim Behav Sci. 2007;107(1–2):100–9. 10.1016/j.applanim.2006.08.002 WOS:000250019700010.

[19] Makowska IJ, Niel L, Kirkden RD, Weary DM. Rats show aversion to argon-induced hypoxia. Appl Anim Behav Sci. 2008;114(3–4):572–81. 10.1016/j.applanim.2008.04.005 WOS:000260706600021.

[20] Boivin GP, Hickman DL, Creamer-Hente MA, Pritchett-Corning KR, Bratcher NA. Review of CO(2) as a Euthanasia Agent for Laboratory Rats and Mice. J Am Assoc Lab Anim Sci. 2017;56(5):491–9. 28903819 PMC5605172

[21] Dryden R, Morgan MG, Bostrom A, Bruine de Bruin W. Public Perceptions of How Long Air Pollution and Carbon Dioxide Remain in the Atmosphere. Risk Anal. 2018;38(3):525–34. 10.1111/risa.12856 . 28666078

[22] Hawkins P, Playle L, Golledge H, Leach M, Banzett R, Coenen AM, et al. Newcastle Consensus Meeting on Carbon Dioxide Euthansia of Laboratory Animals London, UK2006 [cited 2013 7th July 2013]. Available from: https://www.nc3rs.org.uk/sites/default/files/documents/Events/First%20Newcastle%20consensus%20meeting%20report.pdf.

[23] Leary S, Underwood W, Anthony R, Cartner S, Corey D, Grandin T, et al. AVMA Guidelines for the Euthanasia of Animals: 2013 Edition 2013 [cited 2013 29.07.2013]. Available from: works.bepress.com/cheryl_greenacre/14/.

[24] Hewett TA, Kovacs MS, Artwohl JE, Bennett BT. A comparison of euthanasia methods in rats, using carbon dioxide in prefilled and fixed flow rate filled chambers. Lab Anim Sci. 1993;43(6):579–82. . 8158983

[25] Leach MC, Bowell VA, Allan TF, Morton DB. Degrees of aversion shown by rats and mice to different concentrations of inhalational anaesthetics. Vet Rec. 2002;150(26):808–15. Epub 2002/07/18. . 12120924 10.1136/vr.150.26.808

[26] Guedes SR, Valentim AM, Antunes LM. Mice aversion to sevoflurane, isoflurane and carbon dioxide using an approach-avoidance task. Appl Anim Behav Sci. 2017;189:91–7. 10.1016/j.applanim.2017.01.012 WOS:000399625800012.

[27] Niel L, Stewart SA, Weary DA. Effect of flow rate on aversion to gradual-fill carbon dioxide exposure in rats. App Animal Behav Sci. 2008;109(1):77–84.

[28] Chen X, Gallar J, Pozo MA, Baeza M, Belmonte C. CO2 stimulation of the cornea: a comparison between human sensation and nerve activity in polymodal nociceptive afferents of the cat. Eur J Neurosci. 1995;7(6):1154–63. . 7582088 10.1111/j.1460-9568.1995.tb01105.x

[29] Thurauf N, Hummel T, Kettenmann B, Kobal G. Nociceptive and reflexive responses recorded from the human nasal mucosa. Brain Res. 1993;629(2):293–9. . 8111632 10.1016/0006-8993(93)91333-n

[30] Boivin GP, Bottomley MA, Dudley ES, Schiml PA, Wyatt CN, Grobe N. Physiological, Behavioral, and Histological Responses of Male C57BL/6N Mice to Different CO2 Chamber Replacement Rates. J Am Assoc Lab Anim Sci. 2016;55(4):451–61. 27423153 PMC4943617

[31] Powell K, Ethun K, Taylor DK. The effect of light level, CO2 flow rate, and anesthesia on the stress response of mice during CO2 euthanasia. Lab Anim (NY). 2016;45(10):386–95. 10.1038/laban.1117 . 27654690

[32] Anisimov VN, Herbst JA, Abramchuk AN, Latanov AV, Hahnloser RH, Vyssotski AL. Reconstruction of vocal interactions in a group of small songbirds. Nat Methods. 2014;11(11):1135–7. 10.1038/nmeth.3114 . 25262206

[33] Castelhano-Carlos MJ, Baumans V. The impact of light, noise, cage cleaning and in-house transport on welfare and stress of laboratory rats. Lab Anim. 2009;43(4):311–27. 10.1258/la.2009.0080098 . 19505937

[34] de Chaumont F, Coura RD, Serreau P, Cressant A, Chabout J, Granon S, et al Computerized video analysis of social interactions in mice. Nat Methods. 2012;9(4):410–7. 10.1038/nmeth.1924 . 22388289

[35] Thomas AA, Flecknell PA, Golledge HD. Combining nitrous oxide with carbon dioxide decreases the time to loss of consciousness during euthanasia in mice—refinement of animal welfare? PLoS One. 2012;7(3):e32290 10.1371/journal.pone.0032290 22438874 PMC3305278

[36] Hwang E, Kim S, Shin HS, Choi JH. The forced walking test: a novel test for pinpointing the anesthetic-induced transition in consciousness in mouse. J Neurosci Methods. 2010;188(1):14–23. 10.1016/j.jneumeth.2010.01.028 . 20117136

[37] Gelegen C, Gent TC, Ferretti V, Zhang Z, Yustos R, Lan F, et al Staying awake—a genetic region that hinders alpha2 adrenergic receptor agonist-induced sleep. The European journal of neuroscience. 2014;40(1):2311–9. 10.1111/ejn.12570 24674448 PMC4215598

[38] Franks NP. General anaesthesia: from molecular targets to neuronal pathways of sleep and arousal. Nat Rev Neurosci. 2008;9(5):370–86. 10.1038/nrn2372 . 18425091

[39] Kramis R, Vanderwolf CH, Bland BH. Two types of hippocampal rhythmical slow activity in both the rabbit and the rat: relations to behavior and effects of atropine, diethyl ether, urethane, and pentobarbital. Exp Neurol. 1975;49(1 Pt 1):58–85. . 1183532 10.1016/0014-4886(75)90195-8

[40] Sainsbury RS, Heynen A, Montoya CP. Behavioral correlates of hippocampal type 2 theta in the rat. Physiol Behav. 1987;39(4):513–9. Epub 1987/01/01. 0031-9384(87)90382-9 [pii]. . 3575499 10.1016/0031-9384(87)90382-9

[41] Vanderwolf CH. Hippocampal electrical activity and voluntary movement in the rat. Electroencephalogr Clin Neurophysiol. 1969;26(4):407–18. . 4183562 10.1016/0013-4694(69)90092-3

[42] Brankack J, Kukushka VI, Vyssotski AL, Draguhn A. EEG gamma frequency and sleep-wake scoring in mice: comparing two types of supervised classifiers. Brain Res. 2010;1322:59–71. 10.1016/j.brainres.2010.01.069 . 20123089

[43] Langford DJ, Bailey AL, Chanda ML, Clarke SE, Drummond TE, Echols S, et al Coding of facial expressions of pain in the laboratory mouse. Nat Methods. 2010;7(6):447–9. 10.1038/nmeth.1455 . 20453868

[44] Wahlsten D. Phenotypic and genetic relations between initial response to electric shock and rate of avoidance learning in mice. Behav Genet. 1972;2(2):211–40. . 4664210 10.1007/BF01065691

[45] van Rijn CM, Krijnen H, Menting-Hermeling S, Coenen AM. Decapitation in rats: latency to unconsciousness and the 'wave of death'. PLoS One. 2011;6(1):e16514 Epub 2011/02/10. 10.1371/journal.pone.0016514 . 21304584 PMC3029360

[46] Moody CM, Weary DM. Mouse aversion to isoflurane versus carbon dioxide gas. Appl Anim Behav Sci. 2014;158:95–101. 10.1016/j.applanim.2014.04.011 WOS:000341480200011.

[47] Shusterman D, Balmes J. Documentation of nasal irritant sensitivity utilizing pulsed carbon dioxide stimuli. J Allergy Clin Immun. 1996;97(1):86–. WOS:A1996TV53600086.

[48] Kaur S, Wang JL, Ferrari L, Thankachan S, Kroeger D, Venner A, et al A Genetically Defined Circuit for Arousal from Sleep during Hypercapnia. Neuron. 2017 10.1016/j.neuron.2017.10.009 . 29103805 PMC5720904

[49] Qiu MH, Chen MC, Fuller PM, Lu J. Stimulation of the Pontine Parabrachial Nucleus Promotes Wakefulness via Extra-thalamic Forebrain Circuit Nodes. Curr Biol. 2016;26(17):2301–12. 10.1016/j.cub.2016.07.054 27546576 PMC5025760

[50] Mozhui K, Karlsson RM, Kash TL, Ihne J, Norcross M, Patel S, et al Strain differences in stress responsivity are associated with divergent amygdala gene expression and glutamate-mediated neuronal excitability. J Neurosci. 2010;30(15):5357–67. 10.1523/JNEUROSCI.5017-09.2010 20392957 PMC2866495

[51] Okitsu Y, Nehashi S, Iiyori N, Nishino T. Respiratory and behavioural compensation during chronic severe loading in a hypoxic rat model. Clin Exp Pharmacol Physiol. 2004;31(1–2):14–21. 14756679 10.1111/j.1440-1681.2004.03944.x

[52] Benedetti F, Durando J, Giudetti L, Pampallona A, Vighetti S. High-altitude headache: the effects of real vs sham oxygen administration. Pain. 2015;156(11):2326–36. 10.1097/j.pain.0000000000000288 . 26164587

