# Supplementary figures and images for: Nitrogen gas produces less behavioural and neurophysiological excitation than carbon dioxide in mice undergoing euthanasia
Source: PLoS One. 2019 Jan 31;14(1):e0210818. doi: 10.1371/journal.pone.0210818 (PMC6354991; doi:10.1371/journal.pone.0210818)

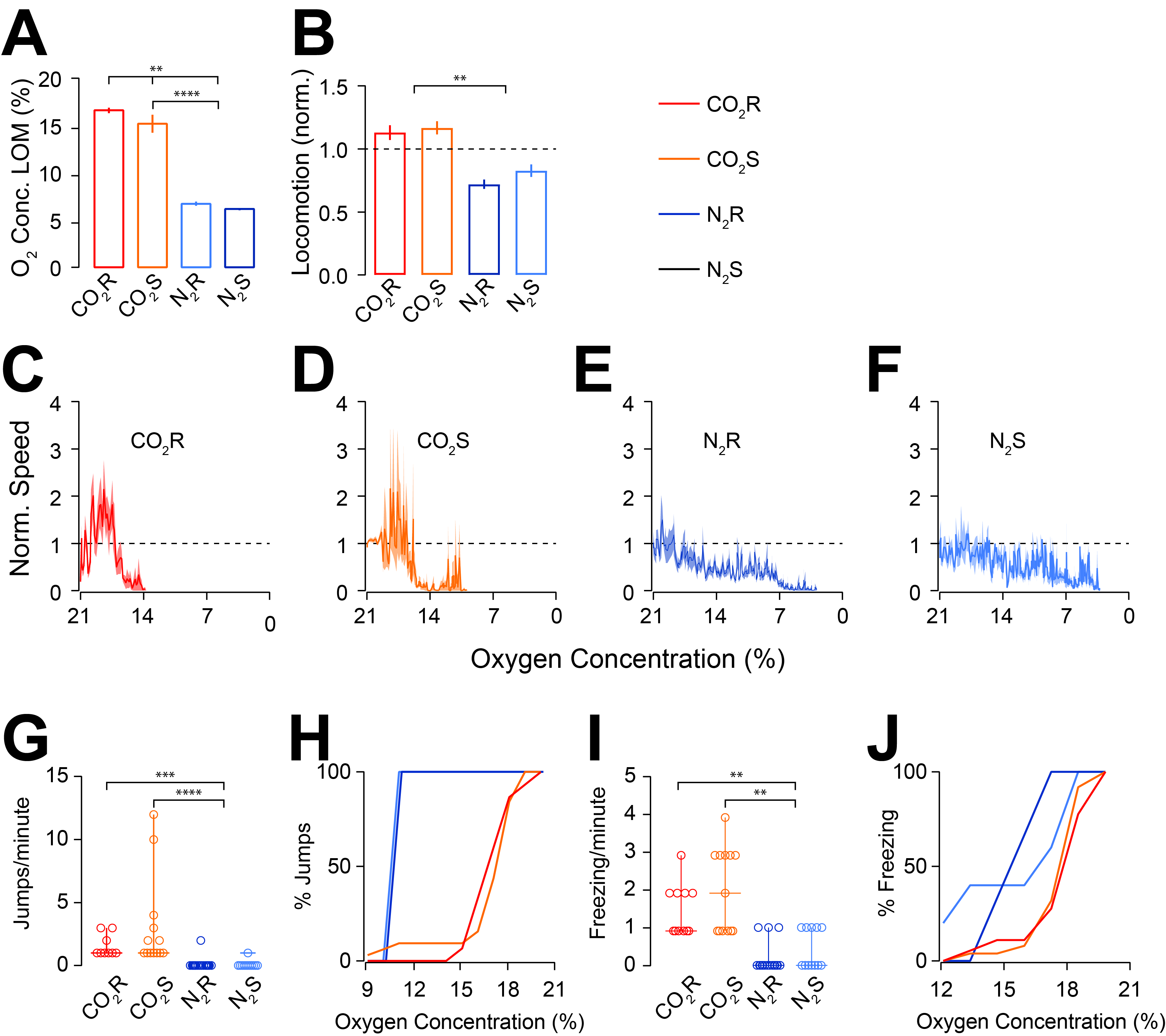

Supplement: S1 Fig — Behavioural markers in the four treatment groups: CO2 rapid fill (CO2R), CO2 slow fill (CO2S), nitrogen rapid fill (N2R) and nitrogen slow fill (N2S). (A) Average (mean ± s.e.m) oxygen concentration at loss of motion (LOM). CO2R resulted in LOM at higher oxygen concentrations than other groups. CO2S resulted in LOM at oxygen concentrations higher than nitrogen groups (**P < 0.01; ****P < 0.0001). (B) Average speed (mean ± s.e.m), normalised to the last 30 seconds of baseline. Horizontal line indicates the normalisation value for baseline measurements. CO2 resulted in increased locomotion compared to nitrogen (**P < 0.01). (C-F) Normalised speed with respect to oxygen concentration from the start of gas exposure until LOM for CO2R (C), CO2S (D), N2R (E), and N2S (F). (G) Vertical jumps shown as median of jumps per animal and inter-quartile range per cage. Both CO2R and CO2S resulted in significantly more jumps compared to N2S (***P < 0.001; ****P < O.0001) (H) Cumulative curves indicating the relative number of jumps in relation with oxygen concentration of the four treatment groups. (I) Freezing shown as mean (± s.e.m) number of freezing episodes per minute per animal. Mice in CO2S froze significantly more times than in the other three groups (**P < 0.01). (J) Cumulative curves indicating the relative freezing episodes in relation with the oxygen concentration of the four treatment groups. (TIF) [file pone.0210818.s001.tif]
